# The effect of pre-emptive oral pregabalin on opioid consumption in patients undergoing laparoscopic sleeve gastrectomy with an analysis of intraoperative hemodynamic stability and quality of recovery: study protocol for a randomized, prospective, double-blind study

**DOI:** 10.1186/s13063-024-08225-3

**Published:** 2024-06-07

**Authors:** Piotr Mieszczanski, Grzegorz Gorniewski, Marek Janiak, Janusz Trzebicki

**Affiliations:** 1St Department of Anesthesiology and Intensive Care, Medical University of Warsaw, Lindleya 4, Warsaw, 02-005 Poland

**Keywords:** Pregabalin, Sleeve gastrectomy, Multimodal analgesia, Quality of recovery

## Abstract

**Background:**

Obese patients undergoing laparoscopic sleeve gastrectomy (LSG) are particularly at risk of opioid-related side effects. To reduce patient exposure to opioids, multimodal analgesia, which involves the use of drugs of different classes, may be utilized. One of the drugs under consideration is pregabalin. Despite an opioid-sparing potential, few studies assess the role of pregabalin as an element of multimodal analgesia in LSG. Considering the limited number and inconsistent results of available studies, we decided to conduct a randomized, prospective study on the effect of preemptive pregabalin administration in obese patients on opioid consumption, pain scores, the incidence of opioid side effects, and hemodynamical stability.

**Methods:**

The study is designed as a prospective randomized controlled trial with double-blinding. Randomization will be performed in a block with a parallel 1:1 allocation. The intervention will involve receiving a pregabalin 150 mg capsule 1–2 h before the surgery, whereas the control group will receive an identically looking placebo. The primary outcome measure will be total oxycodone consumption in the first 24 h following surgery. Secondary outcome measures will be pain severity assessed using the Numerical Rating Scale (NRS) 1, 6, 12, and 24 h after surgery, postoperative sedation on the Ramsay scale, PONV impact scale, the incidence of desaturation episodes < 94%, and episodes of blurred vision at 1, 6, 12, and 24 h after surgery, intraoperative hemodynamic parameters such as heart rate (HR), systolic blood pressure (SBP), diastolic blood pressure (DBP), mean blood pressure (MBP), total fluid volume, and total ephedrine dose. Patient comfort will be additionally assessed using the QoR-40 questionnaire at discharge.

**Discussion:**

The study will explore the efficacy and safety of preemptive pregabalin in a dose of 150 mg as a co-analgesic used in multimodal analgesia for LSG. As studies on opioid-sparing regimes concern the safety of obese patients, we aim to contribute objective data with a relatively large study sample size. The result of the present clinical trial may support the reassessment of recommendations to use pregabalin in the studied population.

**Trial registration:**

ClinicalTrials.gov NCT05804591. Registered on 07.04.2023.

**Supplementary Information:**

The online version contains supplementary material available at 10.1186/s13063-024-08225-3.

## Administrative information

Note: the numbers in curly brackets in this protocol refer to the SPIRIT checklist item numbers. The order of the items has been modified to group similar items (see http://www.equator-network.org/reporting-guidelines/spirit-2013-statement-defining-standard-protocol-items-for-clinical-trials/).

**Table:** 

Title {1}	The effect of pre-emptive oral pregabalin on opioid consumption in patients undergoing laparoscopic sleeve gastrectomy with an analysis of intraoperative hemodynamic stability and quality of recovery: study protocol for a randomized, prospective, double-blind study
Trial registration {2a and 2b}	ClinicalTrials.gov (NCT05804591) Date of registration 07.04.2023 All items from the WHO Trial Registration Dataset can be found on the ClinicalTrials.gov
Protocol version {3}	7th April 2023 Protocol version 1.0
Funding {4}	This research received no external funding
Author details {5a}	Piotr Mieszczanski, Grzegorz Gorniewski, Marek Janiak, Janusz Trzebicki 1st Department of Anesthesiology and Intensive Care, Medical University of Warsaw, Lindleya 4, 02–005 Warszawa, Poland + 482,250,221,721 klinanest1@wum.edu.pl
Name and contact information for the trial sponsor {5b}	Medical University of Warsaw 1st Department of Anesthesiology and Intensive Care, Lindleya 4, 02–005 Warszawa, Poland. + 482,250,221,721 klinanest1@wum.edu.pl
Role of sponsor {5c}	The sponsor has no role in the study design, data collection, or publication

### Background and rationale {6a}

Multimodal analgesia is a technique involving different mechanisms of action, owing to which it is possible to reduce or even eliminate the intraoperative use of opioids and significantly decrease their use postoperatively. To achieve this, several co-analgesics, such as alpha-2 agonists, lidocaine, ketamine, magnesium sulfate, and gabapentinoids, are utilized. They are all part of a concept of multimodal analgesia based on addressing different pain mechanisms. The use of multimodal analgesia reduces opioid-induced side effects in the postoperative period, which is especially beneficial for obese patients with a BMI > 35 qualified for laparoscopic bariatric surgery [1, 2]. Such patients are prone to side effects of opioids, primarily respiratory complications, excessive sedation, and a high risk of postoperative nausea and vomiting, which prevent early patient recovery [3].

Pregabalin, one of the drugs used in multimodal analgesia, is a gamma-aminobutyric acid analog. It has anxiolytic, analgesic, and opioid-sparing properties and is commonly used as a first-line treatment for neuropathic pain [4]. Furthermore, it has effectively prevented opioid-induced hyperalgesia [5, 6]. These properties may prove useful in laparoscopic bariatric surgery, during which there is a risk of nerve fiber injury secondary to cutting and coagulation.

The 2018 ESRA procedure-specific postoperative pain management (PROSPECT) recommendations suggest the use of pregabalin in patients who cannot receive simple analgesics [7]. The above statement is based on two trials involving pregabalin perioperatively in patients who underwent LSG. In the study by Schulmeyer et al., a single 150 mg dose of pregabalin 2 h before surgery allowed for a decrease in the total dose of opioids administered in the postoperative period by 50%. What is essential, pregabalin did not increase the rate of experienced side effects such as excessive sedation or dizziness [8]. Nonetheless, this study has significant limitations, as the study did not implement Patient-Controlled Analgesia (PCA) and more importantly, the authors of the PROSPECT recommendations underline the lack of multimodal analgesia in both the study and control groups. In a study performed by Salama et al. [9], a 68% decrease in the total dose of opioids was possible with 75 mg of pregabalin and a dexmedetomidine infusion of 0.4 μg/kg/h. However, in this study, it is impossible to distinguish between the effects of both medications, as dexmedetomidine has also been proven to have analgesic potential. Similar difficulty in assessing the isolated pregabalin effect has been reported in the observational study by Lam et al. In their study, pregabalin, 150 or 300 mg, depending on the patient's weight, was given as an element of multimodal analgesia to reduce or eliminate total postoperative opioid use [10]. In another trial concerning pregabalin, Alimian et al. demonstrated a reduced incidence of PONV with concomitant lower pain scores throughout the postoperative period in patients undergoing laparoscopic gastric bypass surgery [11]. The limitations of this study include a lack of assessment of sedation and the effects of specific components of multimodal analgesia, which can vary depending on the procedure and may differ between types of surgeries; thus, they are not fully generalizable to patients undergoing LSG [12].

Concerning analgesic management in our study, remifentanil is a basic intraoperative opioid due to its rapid elimination and short time of action, which is in concordance with ERAS guidelines [1]. Moreover, it has been proven that these two drugs have a synergistic effect, which may be beneficial in the perioperative period but, on the other hand, may also cause an increased risk of adverse effects [13, 14]. Pregabalin also has an antihyperalgesic effect, possibly attenuating opioid-induced hyperalgesia sparked by remifentanil [6].

In addition, as pregabalin is a promising element of multimodal analgesia strategy, we plan to measure intraoperative parameters relating to patient hemodynamical stability such as heart rate (HR), systolic blood pressure (SBP), diastolic blood pressure (DBP), and mean blood pressure (MBP) every 5 min as well as cumulative doses of vasopressors needed, atropine used to treat bradycardia and administered fluid volume. In our study, we hypothesized that pregabalin might have little or no effect on hemodynamical stability in contrast with most commonly used co-analgesics such as lidocaine, dexmedetomidine, or magnesium sulfate [1, 15–18]. Its possibly negligent impact on the circulatory system would be beneficial as obese patients undergoing LSG are particularly prone to hemodynamic disturbances [19, 20], and hypotension may in these patients spark complications like myocardial infarction or kidney failure [21].

To our knowledge, there are no known studies assessing the impact of pregabalin on patient recovery after LSG, with a specific focus on its sedative effects, in isolation from other medications such as dexmedetomidine [10, 11]. As we hypothesized that pregabalin may have a beneficial impact in this field, such an effect will be measured in the postoperative period on both an objective scale and by filling out the Quality of Recovery-40 questionnaire (QoR-40), constructed to measure patient’s experience after a broad spectrum of surgeries [22].

In conclusion, considering the limited number and inconsistent results of available studies on the effect of preemptive pregabalin administration in obese patients on opioid consumption, pain scores, the incidence of opioid side effects, and hemodynamical stability, we decided to conduct our randomized, prospective, double-blind study.

### Objectives {7}

Our study aims to assess, in the patients with obesity undergoing LSG, what is the difference in total oxycodone consumption (applied by the PCA pump) between preemptive oral pregabalin 150 mg administration compared with placebo, 24 h after the operation. We hypothesized that the investigated intervention would reduce opioid use and improve recovery with potentially fewer opioid side effects, as well as provide similar intraoperative hemodynamical stability.

### Trial design {8}

The study is designed as a double-blind, randomized superiority trial. Equal, parallel 1:1 randomization will be performed using http://www.randomization.com (Dallal GE).

## Methods: participants, interventions, and outcomes

### Study setting {9}

Academic Hospital in Warsaw, Poland.

### Eligibility criteria {10}

Eligible patients should have a BMI > 40 or > 35 with comorbidities, be 18 to 65 years old, and be LSG-eligible. Patients aged above 65 years are rarely qualified for LSG, and the elderly have a higher risk of unwanted effects [23]. Patients who did not agree to participate in the study, are undergoing revision surgery, have an allergy to any of the drugs used in the protocol, have end-stage organ failure, are unable to cooperate in assessing pain intensity on the numerical rating scale (NRS) scale or use a PCA pump will be excluded from the study.

### Who will take informed consent? {26a}

The consent will be taken by one of the four dedicated investigators, trained before by the principal investigator**.** The approach for consent will be made in the hospital 1 day before the scheduled surgery. One of the investigators will provide the potential participant with a description of the study, potential risks, their rights as a participant, other relevant details and take informed, written consent on a prepared consent form. They will also hand the participant information leaflet. At the time of obtaining the consent for study inclusion, the patient will have a chance to ask questions.

### Additional consent provisions for collection and use of participant data and biological specimens {26b}

No blood samples will be obtained in our study. All participants should give informed, written consent to the research team to share relevant data with researchers taking part in the research, as well as regulatory authorities. This information will be explained to participants and made available on the consent form. All participants should agree to the above.

## Interventions

### Explanation for the choice of comparators {6b}

Participants will be randomized into two groups: pregabalin and control. They will receive identically looking capsules 1–2 h before the operation. According to Enhanced Recovery After Bariatric Surgery (ERABS) protocols or European Society of Regional Anaesthesia and Pain Therapy (ESRA) guidelines, no pharmacological agent should be compared to the test drug [7]. Therefore, we will choose a placebo as a comparator. As the sedative effect of pregabalin is dose-related, and a dose of 300 mg may produce a clinically relevant level of sedation, we will investigate a lower dose of 150 mg [1, 7, 24].

The intervention and placebo are produced in our hospital pharmacy department by dedicated hospital pharmacists and trained pharmaceutical technicians. The original capsule containing pregabalin is dismantled and the drug is placed in the capsule used for our trial, identically looking for the intervention and placebo group. Lactose is used as a standard excipient in both groups. Therefore, it is not possible to discern the capsules on appearance or taste. The capsules are prepared in a dedicated room, with temperature, humidity, and light conditions complying with the requirements for drug manufacturing and storage**.** To ensure the quality of the capsules during their manufacturing, the weight of the capsule fill is monitored, and a visual inspection is performed.

### Intervention description {11a}

The Pregabalin group will receive a capsule containing 150 mg pregabalin as a single dose 1–2 h before the surgery, whereas the control group will receive a same-looking capsule with a placebo. Lactose will be used as a standard excipient in all capsules. The capsule composition does not include dyes, preservatives, or additives, guaranteeing a standard, identical appearance. The capsule has a volume of 0.36 ml, ensuring ease of swallowing. The expiratory date is 1 month after production by our hospital pharmacy.

### Criteria for discontinuing or modifying allocated interventions {11b}

Patients will be free to withdraw from the study at request at any time. Theoretically, in rare cases, it is possible that after randomization and receiving the placebo or intervention, the patient would be disqualified from the surgery or anesthesia due to some impossible-to-predict medical factors that would be revealed immediately before the scheduled operation or will not be able to complete the study treatment. In such a case, they would be withdrawn from the “as treated” analysis of the study outcomes.

### Strategies to improve adherence to interventions {11c}

The intervention will be administered to the participant only once during the hospital stay, and this fact is noted in their individual medication chart. Therefore, participants’ adherence to interventions will be assured.

### Relevant concomitant care permitted or prohibited during the trial {11d}

Patients will continue their concomitant treatment due to chronic diseases in the perioperative period unless it is contraindicated in the planned surgery or anesthesia.

### Provisions for post-trial care {30}

All study participants stay under medical supervision during the study period. Should any severe drug adverse reaction to pregabalin occur, specialist consultations are available. After the trial period, the patients are provided with standard, usual care.

### Outcomes {12}

The primary outcome measure will be total oxycodone consumption 24 h after surgery. Secondary outcome measures will be as follows: pain scores on the NRS scale at 1, 6, 12, and 24 h after surgery, postoperative sedation on the Ramsay scale [25], PONV impact scale [26], the incidence of desaturation episodes < 94% and episodes of blurred vision at 1, 6, 12 and 24 h after surgery, intraoperative heart rate (HR), systolic blood pressure (SBP), diastolic blood pressure (DBP), mean blood pressure (MBP): their highest and lowest values, time of MAP < 65 mmHg, > 90 mmHg, HR < 50 and > 90, total fluid volume, total ephedrine dose and patient's comfort assessed in QoR-40 [22] questionnaire at discharge.

### Participant timeline {13}

The participant timeline is detailed in Table 1.

**Table 1:** The participant timeline

	Study period							
	Enrollment	Allocation	Post-allocation					
Timepoint	− 1 day	Day of surgery	Surgery	1 h	6 h	12 h	24 h	Discharge
Enrollment:								
Eligibility screen	X							
Informed consent	X							
Allocation		X						
Interventions: pregabalin or placebo		X						
Assessment:								
Baseline variables	X	X						
Total oxycodone				- >	- >	- >	X	
The NRS scores				X	X	X	X	
Ramsay Score				X	X	X	X	
PONV-Impact score				X	X	X	X	
SatO2 < 94%				X	X	X	X	
Blurred vision				X	X	X	X	
Intraoperative SBP, DBP, MBP and HR			X					
Total fluid			X					
Total ephedrine			X					
QoR-40								X

Enrolment — 1 day before the operation, intervention — 1 to 2 h before surgery, assessments — 24 h postoperatively

### Sample size {14}

The primary outcome of the study is 24-h oxycodone consumption. The mean 24-h oxycodone consumption in our previous study was 31.31 mg in patients on multimodal anesthesia [18] and SD was 13.7. In order to calculate sample size, we made the following assumptions: type 1 error (*α*) was set at 0.05; type 2 error (*β*) at 0.9 based on two-tailed testing. We considered a difference between groups (*δ*) greater than 10 mg, this being roughly 30% of the mean dose given above, and a standard maximal single dose of oxycodone in an adult patient, to be clinically significant. Using a sample size formula for two-tailed testing recommended in [27], a sample size of 76 should be enough to detect a substantial difference, as stated above. Taking into account an assumed mean drop-out of 15%, we have adopted a rounded-up sample size of 90 patients.

### Recruitment {15}

Patient recruitment starts in April 2023 and is planned to end before April 2025. There will be 4 dedicated investigators responsible for the screening and recruitment of potential participants. All patients qualified for the primary laparoscopic sleeve gastrectomy are identified, screened, and approached if they meet inclusion and do not have exclusion criteria. All patients, prior to study inclusion and signing the consent forms, are reassured that their participation in this trial is entirely voluntary and that refusing to participate or withdrawal at any time during the study would not result in any kind of penalty or negative consequences for the patient.

## Assignment of interventions: allocation

### Sequence generation {16a}

The randomization sequence is based on http://www.randomization.com (Dallal GE) performed by an investigator not involved in patient clinical assessment before the start of enrollment to the study.

### Concealment mechanism {16}

The list is generated and accessed by one investigator, who provides the list to the hospital pharmacy department, where the capsules with drug or placebo are produced. The ward personnel, including the nurse administrating the capsules to the patients, and operating theatre personnel, including anesthesiologists, have no knowledge of patient group allocation.

### Implementation {16c}

All subjects who consent to participate and fulfill the inclusion criteria are randomly assigned to pregabalin or placebo groups. The principal investigator will receive the allocation sequence only after the last participant has completed the trial observation period.

## Assignment of interventions: blinding

### Who will be blinded {17a}

The participants, ward, operating theatre, and postoperative care unit personnel, as well as investigators assessing clinical data of the patients will be blinded to subject allocation.

### Procedure for unblinding if needed {17b}

Contact with the investigator responsible for unblinding is possible at all stages of the study. Their mobile telephone number is on the protocols in which the participant's data is collected. If the principal investigator is unavailable, there is an alternative contact to a second, dedicated researcher. In the event of immediate unblinding of the randomization, contact with the trial methodologist (GG), or on-duty staff of the hospital pharmacy is possible even in out-of-hour time. Unblinding is permissible in case of serious complications or suspected severe adverse reactions with a possible relation to the intervention.

## Data collection and management

### Plans for assessment and collection of outcomes {18a}

Data will be collected in 2 protocols: one for the intraoperative evaluation, filled in by the anesthesiologist, and one dedicated for the postoperative period, filled in by the PACU nurse. The nurses note data, such as oxycodone use from the PCA pump and pain scores (NRS) at specified time points, and record these in a dedicated protocol.

### Plans to promote participant retention and complete follow-up {18b}

Data will be collected during the hospital stay. At the end of the hospital stay, as a standard 24 h after the surgery, study subjects will be encouraged to fill in the QoR-40 questionnaire.

### Data management {19}

The data will be collected in paper form and stored in binders, to which only the principal investigator will have access. After data collection, investigators will check all forms for missing records. The data will be entered manually into an electronic database independently by one investigator, checked for accuracy by a second investigator, and stored on a secure database accessible with a personal login**.** After completion of the study, all data and study documents will be archived and stored by the principal investigator. The data is not public, but upon reasonable request, anonymous data can be made available.

### Confidentiality {27}

The data will be treated anonymously and confidentially, and the personal details of participants will not be revealed at any stage of the study. Every participant receives an ID number to anonymize data collection. The identifiable data will be stored separately in paper form in binders, whereas typed-in, anonymized, unidentifiable data will be stored only in electronic form in a secured database.

### Plans for collection, laboratory evaluation, and storage of biological specimens for genetic or molecular analysis in this trial/future use {33}

No biological specimens will be collected.

## Statistical methods

### Statistical methods for primary and secondary outcomes {20a}

We set the significance level at *α* = 0.05, consistent with common practice in the biomedical sciences, to minimize the probability of a Type I error, which involves incorrectly rejecting a true null hypothesis. The distribution of numerical variables was evaluated for normality using the Shapiro–Wilk test.

#### Statistical methods for numerical outcomes with one-time measurement

For numeric outcomes involving a single measurement point (e.g., total oxycodone consumption at 24 h, comparison of lowest and highest BP and HR values, duration of MBP below 65 mmHg or above 90 mmHg, HR below 50/min, and HR above 90/min, total fluid volume administered, total ephedrine usage during surgery, QoR-40 score at discharge), the significance of differences between the study group and the control group will be assessed using the Wilcoxon sum rank test. This non-parametric test is chosen based on the assumption that the distributions of these numerical variables do not conform to a normal distribution. For each outcome variable, the median and interquartile range (IQR) will be reported for both the study and control groups. In addition to the *p*-values, the Wilcoxon effect size (*r*) will be calculated to quantify the magnitude of the difference between the groups. Where possible, 95% confidence intervals for median differences between groups will be reported to provide an estimate of the precision of the observed effects.

#### Statistical methods for numerical outcomes with multiple measurements

Estimation of the differences between the treatment and control groups for the numerical variables, specifically the NRS score and the PONV-Impact score was conducted using a Repeated Measures Linear Mixed Effects Regression (RLMER) model. This approach was chosen to appropriately handle the intrinsic correlation within patient-level repeated measures data collected at multiple time points. The RLMER model was structured to include fixed effects for the treatment group, time points, and the interaction between the treatment group and time, allowing us to assess how treatment effects vary over time. Additionally, patient-specific random intercepts were incorporated to account for individual variability in baseline scores, which assumes that each patient has a unique starting point that affects all their measurements. We also controlled for potential confounders (e.g. sex, age, BMI) by including them as fixed effects in the model (see Additional file 1: Appendix A for the RLMER model specification).

The effect sizes (Cohen’s *d*) between the treatment and control groups at each specific time point were estimated through contrast analysis, utilizing the estimation of marginal means (EMMs).

#### Statistical methods for dichotomic outcomes with multiple measurements

For the dichotomous outcomes, specifically for instances of SatO2 falling below 94% and the occurrence of blurred vision, differences between the treatment and control groups at each time point were systematically analyzed using a Generalized Linear Mixed Effects Model (GLMER) with a logit link function. This model was chosen to appropriately handle the binary nature of the data, where the outcomes were coded as 1 for events (i.e., SatO2 below 94% or blurred vision) and 0 otherwise. The differences between groups at each time point were quantified using Odds Ratios (ORs), derived from the estimated marginal means (EMMs) of the fitted GLMER model. Odds Ratios represent the odds of the event occurring in the treatment group relative to the control group, adjusted for other model factors. The ORs, along with their 95% confidence intervals and *p*-values, were presented at each time point to assess the strength and significance of the group differences. These results provide insights into how the likelihood of adverse outcomes (low SatO2 or blurred vision) varies between the treatment and control groups across different time points.

#### Statistical methods for ordinal outcomes with multiple measurements

For an ordinal outcome, such as the Ramsay score, cumulative link mixed model (CLMM) also known as proportional odds model was used. The response variable, Ramsay score, was modeled using the proportional odds assumption, where the cumulative log-odds of being at or below a certain category are modeled linearly in terms of predictors (see Additional file 3: Appendix C for model specification). The model estimates provided insights into how the probability of achieving a certain level of sedation changes over time and differs between treatment groups while controlling for other covariates.

#### Characteristics of the statistical tool and external packages

Analyses will be conducted using the R Statistical language (version 4.3.1; R Core Team, 2023) [28] on Windows 10 pro 64 bit (build 19,045), using the packages lme4 (version 1.1. [29]), Matrix (version 1.6.1.1; [30]), robustlmm (version 3.2.3; [31]), emmeans (version 1.8.9; [32]), ggeffects (version 1.3.2; [33]), sjPlot (version 2.8.15; [34]), performance (version 0.10.8; [35]), report (version 0.5.7; [36]) and gtsummary (version 1.7.2; [37]).

As our study is not a high-risk study that uses complex statistical methods, we decided to integrate a statistical analysis plan in this study protocol instead of publishing a separate, detailed statistical analysis plan before the analyses are undertaken [38, 39].

## Data collection and monitoring

Clinical data will be entered into protocols in paper form. After each assessment, the identifiers (e.g., name and birth date) will be anonymized, coded, and stored securely. The files will be backed up in a password-protected database. Data will be handled according to EU and local regulations.

### Interim analyses {21b}

No interim analyses are planned, and no serious adverse effects are expected to arise during the study, as all therapeutic methods are well-established in many other clinical settings.

### Methods for additional analyses (e.g., subgroup analyses) {20b}

As yet, there is no plan to perform subgroup statistical analysis.

### Methods in analysis to handle protocol non-adherence and any statistical methods to handle missing data {20c}

Analyses will be performed for the groups as randomized, primarily with an “as treated” approach. Participants withdrawing from the trial will be followed up, according to the routine clinical practices, but not analyzed further from the point of withdrawal unless they consent using the selected data.

In this type of clinical study, with a very short observation time of clinical data, which is monitored in the PACU, the possibility of missing values will be very low, especially in the primary outcome measure, as the data on oxycodone consumption will be collected using electronic PCA log. Overall, we expect missing outcome data to be minimal and only due to human error or equipment malfunction and, therefore, completely at random. In such a case, missing data will not be replaced.

### Plans to give access to the full protocol, participant-level data and statistical code {31c}

Data associated with published work will be available upon reasonable request. Should this occur, only anonymous data will be made available to protect participant confidentiality.

### Composition of the coordinating center and trial steering committee {5d}

The study will be coordinated by the principal investigator and one dedicated researcher, who will coordinate all phases, including randomization and data storage.

### Composition of the data monitoring committee, its role and reporting structure {21a}

The study includes no interim analysis; the patients involved have non-critical conditions and will undergo treatment for a relatively brief period. Furthermore, pregabalin has a well-established safety profile with a very low probability of harm to the patient. Therefore, apart from the supervision of the Medical University of Warsaw, no external data monitoring is planned.

### Adverse event reporting and harms {22}

Most reported adverse effects caused by pregabalin were mild to moderate intensity, dose-dependent, and occurred within the first 2 weeks of initiating treatment. The most common adverse reactions reported across all patient populations in premarketing controlled trials, which occurred in greater than or equal to 5% of patients taking pregabalin and twice the rate reported by patients receiving placebo, were: somnolence, dizziness, blurred vision, difficulty with concentration/attention, dry mouth, edema, and weight gain [40].

In our trial, participants will be advised to contact ward personnel as soon as possible in case of unexpected or adverse effects or any discomfort supposedly associated with the capsule intake. All the patients will be supervised during their hospital stay, and all possible adverse events or reactions will be observed, recorded, and reported in the study.

### Frequency and plans for auditing trial conduct {23}

The research team will discuss trial conduct during a meeting every 3 months or more frequently if necessary. If there are any changes in the study, the Bioethics Committee, the journal, and Clinical Trials will be notified as soon as possible.

### Plans for communicating important protocol amendments to relevant parties (e.g. trial participants, ethical committees) {25}

This study has been approved by the Bioethics Committee of the Medical University of Warsaw (KB/17/2023), and the study was registered on 07.04.2023 in Clinical Trials (NCT05804591). The study was compliant with the principles outlined in the Declaration of Helsinki and adhered to the applicable CONSORT guidelines.

Patients eligible for recruitment will obtain detailed information about the trial, including potential risks, and subsequently, informed, written consent will be obtained. The consent form was approved by the Bioethics Committee.

Any deviation from the protocol will be documented in a report. All significant protocol modifications have to be reviewed by the Bioethics Committee, then registered in Clinical Trials, and communicated among the researchers. If the participant information changes, updated consent forms and patient leaflets have to be used.

### Dissemination plans {31a}

The trial team will disseminate the results. The team will meet every month to discuss the progress of the study. The results obtained from this study will be disseminated at conferences. A full study report will be submitted for publication in a peer-reviewed journal. We do not plan to notify the participants of the results of the study as a standard, but we can do so upon request.

## Discussion

We describe the protocol of a clinical trial to evaluate the effect of preemptive oral pregabalin administration as an element of multimodal analgesia strategy in patients undergoing LSG, which is most commonly performed in bariatric surgery [41]. Given the limited number of clinical trials and methodological restrictions in existing publications [8–11], which demonstrate varying but significant opioid use reduction in the postoperative period as well as the scarce amount or absence of studies focusing on other significant aspects, additional evidence is required before incorporating pregabalin into a multimodal regimen in the perioperative management of LSG [42].

Our study’s possible limitation may be the use of a *z*-test for the primary outcome to estimate the sample size, while we expect the use of a non-parametric test in the analysis. Therefore, the non-parametric analysis may not reach 90% power. However, as we applied a 15% larger sample size, it may compensate for using a non-parametric test.

In conclusion, the results of the trial based on our protocol will aim at filling this gap and provide us with evidence on the effect of pregabalin administration in a dose of 150 mg on opioid consumption, pain scores, quality of recovery, and hemodynamic stability, which may contribute to a reassessment of recommendations to use this drug in the patients undergoing LSG.

## Trial status

The current protocol version 1.0 is dated 07.04.2023. The recruitment start date is 24th April 2023 and it is planned to be completed by April 2025. Our study is currently enrolling participants.

### Supplementary Information

Additional file 1: Appendix A – The RLMER model specification.

Additional file 2: Appendix B – The GLMER model specification.

Additional file 3: Appendix C – The CLMM model specification.

## Data Availability

The anonymized data generated and analyzed during the current study, including the statistical code, will be available from the corresponding author upon reasonable request.
