# Supplementary material for: The effect of pre-emptive oral pregabalin on opioid consumption in patients undergoing laparoscopic sleeve gastrectomy with an analysis of intraoperative hemodynamic stability and quality of recovery: study protocol for a randomized, prospective, double-blind study
Source: Trials. 2024 Jun 7;25:367. doi: 10.1186/s13063-024-08225-3 (PMC11157713; doi:10.1186/s13063-024-08225-3)
Supplement: Supplementary file 1 — Additional file 1: Appendix A – The RLMER model specification. [file 13063_2024_8225_MOESM1_ESM.pdf]

Appendices

Appendix A – The RLMER model specification

The RLMER model with decomposition for each level:

Level 1: Within-patient (sample level)

The modelling the outcome variable y for each measurement occasion j (time points: 1h, 6h, 12h, 24h) within each patient i. In the equation (1) the model includes fixed effects for time, group, and their interaction, plus patient-specific adjustments (random intercepts).

$$y_{ij} = \pi_{0i} + \pi_{1i}\ (time_{ij}) + \ {}_{ij}\ (1)$$

where π_{0i} is the overall effect for patient i (including the base level of the outcome and the patient-specific deviation captured by the random intercept); $\pi_{1i}\ (time_{ij})$ represents the time-specific effects, adjusted for interactions with groups and confounders; ${}_{ij}$ is the residual error for patient i at time j, assumed iid $N\,(0, \sigma^2)$.

Level 2: Between-Patient

At this level, by the equation (2) we model the random effects and the influence of patient-level predictors (e.g., treatment group, confounders). The intercepts are considered as outcomes influenced by these higher-level predictors.

$$\pi_{0i} = \beta_0 + \beta_4\ group_i + X_i^T\ \beta_X + b_{0i},\ (2)$$

$$\pi_{1i}\,(time_{ij}) = \beta 1 \cdot \delta(time_{ij},\ 6h) + \beta 2 \cdot \delta(time_{ij},\ 12h) + \beta 3 \cdot \delta(time_{ij},\ 24h) +$$

$$+\ \beta 5 \cdot \delta(time_{ij},\ 6h) \times group_i + \beta 6 \cdot \delta(time_{ij},\ 12h) \times group_i + \beta 7 \cdot \delta(time_{ij},\ 24h) \times group_i,\ (3),$$

where $\beta_{0,} \beta_{1,} \beta_{2,} \beta_3$ are the fixed effects coefficients for the baseline and time points; $\beta_{4,} \beta_{5,}$ $\beta_{6,} \beta_7$ are the coefficients for group effects and interaction terms between group and time; δ is the indicator function, 1 if the condition is true and 0 otherwise (a mathematical way to handle categorical variables in equations); X_i – is the vector of confounders for patient I; b_{0i} – is the random intercept for each patient i, assumed $N\,(0, \sigma^2_{b0})$.
