# Supplementary material for: The effect of pre-emptive oral pregabalin on opioid consumption in patients undergoing laparoscopic sleeve gastrectomy with an analysis of intraoperative hemodynamic stability and quality of recovery: study protocol for a randomized, prospective, double-blind study
Source: Trials. 2024 Jun 7;25:367. doi: 10.1186/s13063-024-08225-3 (PMC11157713; doi:10.1186/s13063-024-08225-3)
Supplement: Supplementary file 2 — Additional file 2: Appendix B – The GLMER model specification. [file 13063_2024_8225_MOESM2_ESM.pdf]

Appendix B – The GLMER model specification

Level 1: Sample Level (Within-Patient)

In this level we model the outcome for each measurement occasion within patients. For the outcome for patient *i* at the time *j* y_{ij}, the logit of the expected value of y_{ij} is modeled as a function of predictors by (3)

$$\text{Logit}\,(E[y_{ij}]) = \eta_{ij}\ (3)$$

Where η_{ij} is the linear predictor, defined by (4):

$$\eta_{ij} = \beta_0 + \beta_1\, time_{ij} + \beta_4\, group_i + \beta_3\, (time_{ij} \times group_i) + X_i\gamma + u_i,\ (4)$$

where β_0 represents the global intercept; β_1 represents the coefficients for the time effect (with time treated as categorical for 1h, 6h, 12h, 24h); β_2 is the coefficient for the effect for the interaction between time and group, capturing how the effect of the group varies at different time points; $X_i\gamma$ represents the fixed effects of confounders, where X_i is a vector of confounder variables for the patient *i* and γ is a vector of coefficients; u_i – is the random intercept for patient *i*, accounting for the intra-patient correlation across repeated measures.

Level 2: Patient level

At this level the random effects are specified by (5):

$$u_i \sim N\,(0, \sigma_u^2),\ (5)$$

Here, u_i captures the patient-specific deviation from the overall intercept β_0 and σ_u^2 is the variance of these patient-specific random intercepts.
