# Supplementary material for: The effect of pre-emptive oral pregabalin on opioid consumption in patients undergoing laparoscopic sleeve gastrectomy with an analysis of intraoperative hemodynamic stability and quality of recovery: study protocol for a randomized, prospective, double-blind study
Source: Trials. 2024 Jun 7;25:367. doi: 10.1186/s13063-024-08225-3 (PMC11157713; doi:10.1186/s13063-024-08225-3)
Supplement: Supplementary file 3 — Additional file 3: Appendix C – The CLMM model specification. [file 13063_2024_8225_MOESM3_ESM.pdf]

Appendix C – The CLMM model specification

The CLMM model assumes that the relationship between each pair of outcome categories is the same, and it uses a series of binary logistic regressions to model the cumulative probabilities up to each category.

The structure a cumulative link mixed model (CLMM) using a proportional odds assumption, for an ordinal outcome measured at four time points (1h, 6h, 12h, 24h), considering interactions between time and group, along with confounders, and including random intercepts for patients defined by (6), (7).

Level 1: Sample Level (Within-Patient)

For ordinal outcome y_{ij} for patient I at time j and the number of K ordinal categories, the logit of cumulative probability P of y_{ij} being less than or equal to category k is modelled by(6):

$$logit(P(y_{ij} \leq k)) = \alpha_k - (\beta_0 + \beta_1\, time_{ij} + \beta_4\, group_i + \beta_3\, (time_{ij} \times group_i) + X_i\gamma + u_i).\ (6),$$

where α_k are the thresholds (cutpoints) for each category k, which estimated from the data but not associated with any predictor variables. For the rest of the nomenclature, see Appendix B.

Level 2: Patient Level

The patient-specific random effects are specified by (7):

$$u_i \sim N\,(0, \sigma_u^2),\ (5)$$

Similarly to GLMER, the u_i captures the patient-specific deviation from the overall intercept β_0 and σ_u^2 is the variance of these patient-specific random intercepts.
